# How long do natural waters “remember” release incidents of Marcellus Shale waters: a first order approximation using reactive transport modeling

**DOI:** 10.1186/s12932-016-0038-4

**Published:** 2016-12-13

**Authors:** Zhang Cai, Li Li

**Affiliations:** 1 John and Willie Leone Family Department of Energy and Mineral Engineering, The Pennsylvania State University, University Park, PA 16802 USA; 2 Department of Civil and Environmental Engineering, The Pennsylvania State University, University Park, PA 16802 USA

**Keywords:** Reactive transport modeling, Ion exchange, Marcellus Shale waters, Environmental impacts

## Abstract

Natural gas production from the Marcellus Shale formation has significantly changed energy landscape in recent years. Accidental release, including spills, leakage, and seepage of the Marcellus Shale flow back and produced waters can impose risks on natural water resources. With many competing processes during the reactive transport of chemical species, it is not clear what processes are dominant and govern the impacts of accidental release of Marcellus Shale waters (MSW) into natural waters. Here we carry out numerical experiments to explore this largely unexploited aspect using cations from MSW as tracers with a focus on abiotic interactions between cations released from MSW and natural water systems. Reactive transport models were set up using characteristics of natural water systems (aquifers and rivers) in Bradford County, Pennsylvania. Results show that in clay-rich sandstone aquifers, ion exchange plays a key role in determining the maximum concentration and the time scale of released cations in receiving natural waters. In contrast, mineral dissolution and precipitation play a relatively minor role. The relative time scales of recovery τ_rr_, a dimensionless number defined as the ratio of the time needed to return to background concentrations over the residence time of natural waters, vary between 5 and 10 for Na, Ca, and Mg, and between 10 and 20 for Sr and Ba. In rivers and sand and gravel aquifers with negligible clay, τ_rr_ values are close to 1 because cations are flushed out at approximately one residence time. These values can be used as first order estimates of time scales of released MSW in natural water systems. This work emphasizes the importance of clay content and suggests that it is more likely to detect contamination in clay-rich geological formations. This work highlights the use of reactive transport modeling in understanding natural attenuation, guiding monitoring, and predicting impacts of contamination for risk assessment.

## Background

The development of unconventional natural gas in the Marcellus Shale formation has grown rapidly in recent years. Significant concerns arise in parallel due to their possible impacts on water resources. Here Marcellus Shale waters (MSW) are defined as waters from gas wells including both flowback and produced waters. Marcellus Shale waters are typically characterized by high total dissolved solids (TDS, usually >200,000.00 mg/L), elevated concentrations of Br, Cl, major cations (Na, Ca, Mg, K), as well as Ba and Sr, often accompanied by natural occurring radioactive materials [1–4]. Accidental release of MSWs has been reported to occur through impoundments, drilling site discharge, spills, among others [5–7]. Although these major ions are of less environmental concern than toxic metals, their high concentrations can still pose adverse effects on human health. For example, Br may produce bromate through ozonation, a human carcinogen [8]. High Ba concentration can cause muscle weakness and affects blood pressure, nervous and circulatory system [9, 10]. Their release can deteriorate water quality and aquatic ecological systems [6]. In 2013, four northeastern amphibian species have been recorded to be adversely affected by 50–1000 mg/L chloride, suggesting small accidental releases can impede breeding habitats [11]. They are also important indicators of fracking fluid, flowback and produced water, and brine contamination in aquifers or rivers [5, 12, 13].

**Fig. 1:**
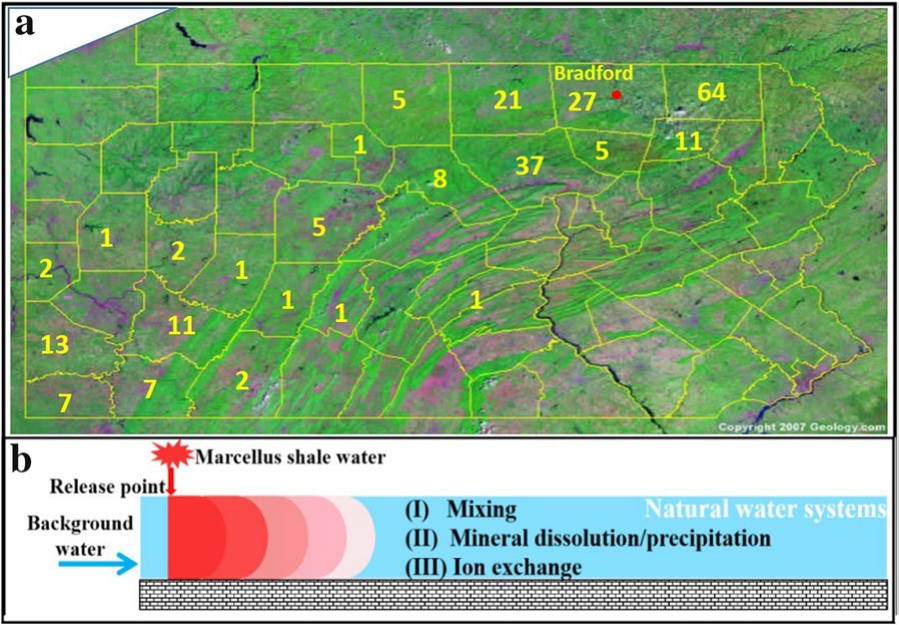
**a** The numbers of Marcellus Shale water release accidents in Pennsylvania from 2005 to June 8, 2015, with 78% of spills occurred in Northeastern PA. *Red spot* indicated the location of Bradford County. The *yellow numbers* are the numbers of spills. **b** A schematic diagram of 1-dimensional modeling setup. We assume a release point where the Marcellus Shale waters are introduced into the surface water (river) or groundwater (aquifers). The release can occur through spills, discharge, leakage, seepage, among others

Recent evidence highlighted the risk of MSW leakage into natural waters. Direct discharge of MSW into surface waters has been frequently reported [14, 15]. In Pennsylvania, a total of 229 spills occurred from 2005 to 2015 [16], as illustrated in Fig. 1a. High concentrations of methane, saline brine [17, 18] and 2-*n*-butoxyethanol (often used in the fracking fluids) [19] were found in drinking groundwater aquifers in Pennsylvania, indicating potential leakage associated with Marcellus Shale gas development. The discharge of MSW has been found to increase downstream Br and Cl concentrations by more than three orders of magnitude [14, 15]. Ferrar et al. [14] found Ba and Sr surpassed the US maximum concentration level (MCL) after a deliberate MSW discharge. Sang et al. [20] reported 32–36% of heavy metals associated with colloids mobilized by flowback water flush.

These studies raise questions regarding the impacts of release incidents. *How long and to what extent do natural waters (rivers and aquifers) “remember” the release of MSW?* In other words, how long do MSW stay in natural waters? The ultimate transport and fate of released chemical species can be affected by many processes (Fig. 1b). Mixing of different waters occur immediately upon release, which means that the relative magnitude of water release rate and the background flow rate in the receiving waters can play a significant role in determining their concentrations [21]. Cations in the Marcellus Shale waters can participate in multiple water–rock interactions, including mineral dissolution and precipitation, ion exchange, and surface complexation when clay minerals are abundant. The geochemical conditions of receiving aquifers, therefore, can be important in determining dominant reactions, natural attenuation potential, and impacts of accidental releases [22]. There has been a significant lack of key measures that quantify and predict reactive transport and fate of chemical species from MSW.

The objective of this study is to (1) understand key processes that govern the reactive transport and fate of major cations from MSW; and to (2) to quantify time scales and magnitude of the release impacts on water quality under various release and receiving water conditions. It is important to note that here we focus on abiotic interactions, instead of microbe-mediated biodegradation of organic contaminants. Heavy metals are not included in this study as they deserve a separate study. The insights learned here can facilitate fundamental understanding of natural attenuation and assess environmental risks. Simulations were done under conditions relevant to natural waters in Bradford County in the Pennsylvania, where local residential concerns on water quality arise in parallel with the large number of drilled wells [23]. We use the multicomponent reactive transport model CrunchFlow [24], which solves conservation equations with respect to mass, momentum, and energy. It has been extensively used to understand and predict reactive transport of contaminants, and water–rock interaction in porous media [25, 26]. To the best of our knowledge, this work is among the early studies that use reactive transport modeling tools to understand the impacts of Marcellus Shale waters in natural water systems.

## Methods

### Problem setup

As shown in Fig. 1b, MSWs are introduced into homogeneous and isotropic natural water systems including ground water in sandstone (S) aquifers and sand and gravel (SG) aquifers and surface water. This represents a base case scenario with major focus on the coupling of transport and geochemical reactions without considering spatial heterogeneities. The interactions between chemical species in MSWs and sediment (typically <2 vol%) in rivers are assumed negligible.

The S aquifers and SG aquifers were chosen as representative aquifers because they dominate in Northeastern Pennsylvania [27]. They differ in mineralogical compositions, with the S aquifers containing much more clay. We chose a branch of the Susquehanna River to represent the river. The release characteristics of MSWs, including release rates, time duration, and therefore total volumes, can vary significantly. All these factors can influence the impacts of accidental release on natural water compositions.

### Properties of natural waters and MSWs

#### Natural water systems

We used the characteristics of a sandstone aquifer with dominant clay mineral of 21.7% in the Catskill Formation in Bradford County, PA. The S aquifer has a groundwater velocity of 0.20 m/day and is predominantly a low-rank graywacke with major minerals being quartz, mica (represented by muscovite) and other clays, and trace amount of carbonate (mostly calcite) [28]. In contrast, the Sand and Gravel aquifer has a groundwater velocity of 0.40 m/day and a lower clay amount than that of the S aquifer [29, 30]. For rivers we choose conditions relevant to the Susquehanna River segment in Bradford County, PA [31], considering 2% (v/v) of suspended sediments [32]. The major difference between the surface and subsurface water systems are the orders of magnitude higher flow rates and the negligible presence of solid phases compared to the aquifers (Table 1).

**Table 1:** Mineral composition and flow velocity in the natural waters

Mineral	Mineral formula	Volume fraction		
		S aquifer [28, 29, 33–35]	SG aquifers [29, 30, 36–39]	River [32, 40–42]
Primary minerals^a^				
Quartz	SiO_2_	4.13 × 10^−1^	5.80 × 10^−1^	6.74 × 10^−3^
K-Feldspar	KAlSi_3_O_8_	3.50 × 10^−1^	1.80 × 10^−1^	7.40 × 10^−4^
Muscovite	KAl_2_(Si_3_Al)O_10_(OH)_2_	1.05 × 10^−1^	0.00	0.00
Sericite	KAl_2_(Si_3_Al)O_10_(OH)_2_	4.20 × 10^−2^	0.00	0.00
Clinochlore-14A	Mg_5_Al_2_(Si_3_O_10_)(OH)_8_	2.80 × 10^−2^	0.00	0.00
Daphnite-14A	Fe_5_Al_2_(Si_3_O_10_)(OH)_8_	2.80 × 10^−2^	0.00	0.00
Kaolinite	Al_2_Si_2_O_5_(OH)_4_	1.40 × 10^−2^	9.00 × 10^−4^	0.00
Illite	K_0.6_Mg_0.25_Al_1.8_(Al_0.5_Si_3.5_O_10_)(OH)_2_	0.00	0.00	1.08 × 10^−2^
Calcite	CaCO_3_	3.50 × 10^−2^	6.00 × 10^−4^	1.50 × 10^−3^
Dolomite	CaMg(CO_3_)_2_	0.00	9.60 × 10^−3^	2.60 × 10^−4^
Suspended sediments	–	**–**	–	2.0 × 10^−2^
Porosity		3.00 × 10^−1^	3.90 × 10^−1^	9.80 × 10^−1^
Total	–	1.00	1.00	1.00
Flow velocity (m/day)		2.00 × 10^−1^ ^b^	4.00 × 10^−1^ ^c^	2.76 × 10^4^ [40–42]
Permeability (m^2^)		5.00 × 10^−13^ ^b^	5.00 × 10^−12^ ^c^	–

^a^Four secondary minerals, including gypsum, celestite, barite, and gibbsite, are initially assigned with a volume fraction of 10^−10^ for precipitation in simulated natural water domain [29, 33]

^b^Porosity and flow velocity are within the typical range for S aquifers in this area [34, 35]

^c^Porosity and flow velocity are within the typical range for SG aquifers [36, 37]

#### Water composition

The three natural waters differ in their chemical composition [38] (Table 2). The surface water has higher concentrations of sulfate and cations including iron, potassium, and zinc, while the ground waters are richer in calcium, magnesium, and sodium. The major difference between the surface and subsurface water systems are the orders of magnitude higher flow rates and the negligible presence of solid phases compared to aquifers. The MSW composition was chosen to be in the low concentration level of produced and flowback waters.

**Table 2:** Composition of natural waters and Marcellus Shale water (mg/L)

Species	S aquifer [38, 43]	SG aquifer [18, 44]	River [45]	Marcellus Shale water [46]
pH	7.40	7.44	7.37	6.90
Br	2.02 × 10^−2^	2. 00 × 10^−2^	1.29 × 10^−2^	1.87 × 10^2^
Cl	7.99 × 10^0^	5.89 × 10^0^	8.20 × 10^0^	2.92 × 10^4^
SO_4_	9.98 × 10^−1^	1.39 × 10^1^	1.54 × 10^1^	6.60 × 10^0^
Al	–	–	–	2.00 × 10^−1^
Ba	1.20 × 10^−1^	1.60 × 10^−1^	2.14 × 10^−1^	1.01 × 10^3^
Cd	–	–	–	4.98 × 10^−2^
Ca	4.24 × 10^1^	3.62 × 10^1^	1.57 × 10^1^	1.59 × 10^3^
Cu	–	–	–	2.50 × 10^−1^
Fe	1.00 × 10^−1^	5.00 × 10^−2^	5.99 × 10^−2^	3.44 × 10^1^
Pb	1.00 × 10^−2^	1.00 × 10^−2^	1.00 × 10^−2^	3.00 × 10^−2^
Mg	1.64 × 10^0^	6.98 × 10^0^	3.07 × 10^0^	1.50 × 10^2^
Mn	6.00 × 10^−3^	6.00 × 10^−3^	–	1.02 × 10^0^
K	2.80 × 10^0^	1.27 × 10^0^	9.01 × 10^−1^	6.40 × 10^2^
Na	1.85 × 10^1^	1.09 × 10^1^	8.37 × 10^0^	1.32 × 10^4^
Sr	2.90 × 10^−1^	2.82 × 10^−1^	–	3.90 × 10^2^
Zn	4.00 × 10^−5^	4.00 × 10^−5^	1.78 × 10^1^	1.70 × 10^−1^
Alkalinity as ${\text{HCO}}_{3}^{ - }$	1.77 × 10^2^ ^a^	1.49 × 10^2^ ^a^	9.88 × 10^1^	2.45 × 10^2^ ^b^

Water chemistry data are among the range of reported literature

^a^Alkalinity (as ${\text{HCO}}_{3}^{ - }$) was calculated based on equilibrium with calcite using CRUNCHFLOW and is in the range of reported value of 51–366 mg/L for sandstone aquifer water and of 85–195 mg/L for sand and gravel aquifer water

^b^Alkalinity is directly from literature. Charges are balanced in all natural waters

### Characteristics of Marcellus Shale water release incident

A total of 9179 unconventional wells were installed in the Marcellus Shale formation in Pennsylvania from 2005 to 2015 [16]. A total of 229 spill accidents have occurred, dictating a spill possibility of 2.40% per well in average. The spill volumes varied from 0.003 to about 11.35 m^3^ with the median value being 0.144 m^3^ [47]. With the same spill volume, a release can occur at small rates for a long duration or high rates for a short time frame. The MSWs reached groundwater by seeping into groundwater aquifers, which is a relatively slow process. Here we assume a net water volume of 0.144 m^3^ reaching natural waters; the actual spill water can be much larger as the vadose zone tends to trap a large percent of spilled water [47]. Here we do not explicit consider vadose zone processes. The spill rates are varied to examine the importance of release characteristics.

We define the dilution factor (DF):

1 $$ DF = \frac{{Q_{MSW} + Q_{NW} }}{{Q_{MSW} }} $$

where Q_MSW_ and Q_NW_ are the volumetric flow rates (m^3^/s) of MSW and the receiving natural waters, respectively. The Q_NW_ values are calculated as the product of flow velocity (m/day) and cross-sectional area of 1 m^2^ in the model. As such, we focus on understanding processes at the immediate vicinity of the leakage point and flow path. The DF quantifies the extent of dilution upon release into natural waters. A high DF value means that the released MSW is quickly diluted by the fast background natural waters. It is important to note here that fluid injection into an aquifer typically only causes limited mixing at the fringes. Here by assuming well-mixed intruding fluid and background water at the injection point, we can use this as a rough estimation of the relatively magnitude of the injection fluid rate versus the background fluid rate.

### Reactive transport modeling

Upon accidental release into natural water systems, the chemical species in the MSWs interact with natural waters and solid phases. Major processes include mixing, transport, and various types of water–rock interactions.

#### Reactive transport equations

Reactive transport models (RTM) have been extensively used to understand complex interactions among physical, chemical, and biological processes in porous media [48–51]. The governing mass conservation equation for a chemical component *i* that participates in ion exchange reactions can be written as follows:

2 $$ \frac{{\partial (\phi C_{i} )}}{\partial t} + \rho \frac{\partial S}{\partial t} = \nabla \cdot \{ \phi {\mathbf{D}}_{{\mathbf{i}}} \nabla (C_{i} ) - \phi {\mathbf{u}}C_{i} \} + \sum\limits_{r = 1}^{Nr} {v_{ir} R_{r} } $$

Here $\phi$ is porosity, C_i_ is total concentration (mol/m^3^ pore volume) of *i*, t is time (s), **D**_**i**_ is diffusion/dispersion tensor (m^2^/s), **u** is flow velocity (m/s), N_r_ is total number of kinetic reactions that involve species *i*, v_ir_ is stoichiometric coefficient of species *i* associated with reaction *r*, R_r_ is the rate of chemical reactions in which the species *i* is involved (mol/m^3^/s). The diffusion/dispersion coefficients and flow velocities are set constant with a dispersivity of 1.0 cm [52]. Here kinetic reactions include mineral dissolution and precipitation. Ion exchange and aqueous complexation are considered as fast and are equilibrium-controlled. This equation implies that mass change rate of species *i* depends on diffusion/dispersion represented by the first term in the right hand side (rhs), advection described by the second term in the rhs, and reaction described by the third term. The term $\rho \frac{\partial S}{\partial t}$ represents mass exchange with solid phase through ion exchange, with ρ being solid bulk density (g/m^3^ pore volume), and *S* being solid phase concentration of *i* (mol/g). This term is essentially a storage term taking into account mass accumulation of *i* on the solid phase [53]. The geochemical system here includes 18 chemical components (Table 2) and 14 kinetic mineral reactions (Table 3).

**Table 3:** Reaction network, Reaction thermodynamics, and kinetics for mineral–water interactions

No.	Minerals	Reactions	log K_eq_ [65]	logk [(mol/m^2^)/s] [69]	SSA^a^
	Kinetic reactions				
1	Quartz	SiO_2_(s) ⇔ SiO_2_(aq)	−4.00	−13.41	0.017 [54]
2	K-Feldspar	KAlSi_3_O_8_ + 4H^+^ ⇔ Al^3+^ + K^+^ + 2H_2_O + 3SiO_2_(aq)	−0.27	−12.41	0.098 [55]
3	Clinochlore-14A	${\text{Mg}}_{5} {\text{Al}}_{2} {\text{Si}}_{3} {\text{O}}_{10} \left( {\text{OH}} \right)_{8} + 8{\text{H}}^{ + } \Leftrightarrow 5{\text{Mg}}^{2 + } + 2{\text{Al}}\left( {\text{OH}} \right)_{4}^{ - } + 3{\text{SiO}}_{2} \left( {\text{aq}} \right) + 4{\text{H}}_{2} {\text{O}}$	67.24	−12.52	1.10 [56]
4	Daphnite-14A	${\text{Fe}}_{5} {\text{Al}}_{2} {\text{Si}}_{3} {\text{O}}_{10} \left( {\text{OH}} \right)_{8} + 8{\text{H}}^{ + } \Leftrightarrow 5{\text{Fe}}^{2 + } + 2{\text{Al}}\left( {\text{OH}} \right)_{4}^{ - } + 3{\text{SiO}}_{2} \left( {\text{aq}} \right) + 4{\text{H}}_{2} {\text{O}}$	52.28	−12.52	1.10 [56]
5	Muscovite	KAl_2_(Si_3_Al)O_10_(OH)_2_ + 10H^+^ ⇔ K^+^ + 3Al^3+^ + 3SiO_2_(aq) + 6H_2_O	13.58	−13.55	14.28 [57]
6	Kaolinite	Al_2_Si_2_O_5_(OH)_4_ + 6H^+^ ⇔ 2Al^3+^ + 5H_2_O + 2SiO_2_	6.81	−13.18	14.70 [58]
7	Illite	K_0.6_Mg_0.25_Al_1.8_Al_0.5_Si_3.5_O_10_(OH)_2_ + 8H^+^ ⇔ 0.25 Mg^2+^+0.6K^+^+2.30Al^3+^ + 3.50SiO_2_(aq) + 5H_2_O	9.02	−11.60	65.00 [57]
8	Sericite	KAl_2_(Si_3_Al)O_10_(OH)_2_ + 10H^+^ ⇔ K^+^ + 3Al^3+^ + 3SiO_2_(aq) + 6H_2_O	13.58	−13.55	57.00 [59]
9	Dolomite	${\text{CaMg}}\left( {{\text{CO}}_{3} } \right)_{2} \left( {\text{s}} \right) \Leftrightarrow {\text{Ca}}^{2 + } + {\text{Mg}}^{2 + } + 2{\text{CO}}_{3}^{2 - }$	−16.70	−7.53	0.25 [60]
10	Calcite	${\text{CaCO}}_{3} \left( {\text{s}} \right) \Leftrightarrow {\text{Ca}}^{2 + } + {\text{CO}}_{3}^{2 - }$	−8.48	−5.81	0.48 [61]
11	Gypsum	${\text{CaSO}}_{ 4} \left( {\text{s}} \right) \Leftrightarrow {\text{Ca}}^{ 2+ } + {\text{SO}}_{4}^{{2{ - }}} + 2 {\text{H}}_{ 2} {\text{O}}$	−4.48	−2.79	7.00 [62]
12	Celestite	${\text{SrSO}}_{ 4} \left( {\text{s}} \right) \Leftrightarrow {\text{Sr}}^{ 2+ } + {\text{SO}}_{4}^{2 - }$	−5.68	–	1.22 [63]
13	Barite	${\text{BaSO}}_{ 4} \left( {\text{s}} \right) \Leftrightarrow {\text{Ba}}^{ 2+ } + {\text{SO}}_{4}^{2 - }$	−9.97	−7.90	1.47 [61]
14	Gibbsite	Al(OH)_3_(s) + 3H^+^ ⇔ Al^3+^ + 3H_2_O	8.11	−11.50	6.50 [64]

No.	Ion exchange	Cation exchange capacity (CEC) [66]		logK [67, 68]		
	(Vanselow)	S aquifer	SG aquifers			
1	Na*X* ⇔ Na^+^ + *X* ^−^	5.0 × 10^−5^ eq/g	3.0 × 10^−5^ eq/g	0.00		
2	K*X* ⇔ K^+^ + *X* ^−^			−0.69		
3	Ca*X*_2_ ⇔ Ca^2+^ + 2*X* ^−^			−0.39		
4	Mg*X*_2_ ⇔ Mg^2+^ + 2*X* ^−^			−0.30		
5	Ba*X*_2_ ⇔ Ba^2+^ + 2*X* ^−^			−0.45		
6	Sr*X*_2_ ⇔ Sr^2+^ + 2*X* ^−^			−0.45		

^a^SSA values are from the laboratory studies in the literature which are generally observed to be faster than those from the fields [55, 70]

The RTM was implemented within a 10 m one-dimensional domain with 100 grid cells and a fixed spatial discretization of 0.1 m. The spatial discretization was chosen as the lowest one that results in the same modeling output as those from spatial resolutions higher than 0.1 m. The injection point is the first grid cell. As such, we are simulating the first 10 m immediately down gradient of an injection point. We choose not to do numerical experiments in a large spatial domain of kilometers because the goal here is to understand dominant geochemical processes that govern natural attenuation of Marcellus Shale waters. A domain of 10 m is sufficient for such purpose. As will be discussed later, the dimensionless time derived from this work is not confined to the physical length of simulated domain. Running simulation at large spatial scales however presents additional challenges, largely because reaction parameters in literature are mostly measured in relatively small scale laboratory systems at the spatial scale of 10^0^–10^2^ cm. Reaction parameters, in particular reaction kinetic constants and effective surface areas, are often orders of magnitude lower in large scale heterogeneous systems [49, 55, 71, 72]. Running simulations at the scale of kilometers therefore requires overcoming upscaling of reaction processes, which has been a long-standing and unresolved puzzle [73, 74].

We examined five cases with different types of water systems and release characteristics (Table 4). The three release rates were determined by using reported dilution factors in literature [47, 75, 76] and Eq. (1).

**Table 4:** Simulation scenarios for Marcellus Shale water release

Receiving water systems	Release rate (m^3^/s)	Release duration (days)	Dilution factor (DF)^a^	Residence time (days)
Sandstone aquifer	1.11 × 10^−8^	1.50 × 10^2^	2.09 × 10^2^	1.50 × 10^1^
	5.55 × 10^−8^	3.00 × 10^1^	4.27 × 10^1^	1.50 × 10^1^
	1.11 × 10^−7^	1.50 × 10^1^	2.18 × 10^1^	1.50 × 10^1^
Sand and gravel aquifers	1.11 × 10^−7^	1.50 × 10^1^	4.27 × 10^1^	9.75 × 10^0^
River	1.11 × 10^−7^	1.50 × 10^1^	2.87 × 10^6^	3.55 × 10^−4^

^a^Values are from literature [47, 75, 76]

#### Mineral dissolution and precipitation

Mineral reactions are listed in Table 3 with their equilibrium constants and reaction kinetics. In the systems in this paper, most waters are at close to neutral conditions so we only use rate laws based on neutral mechanisms and follow the classical transition-state-theory-based (TST) rate law [77]:

3 $$ R_{Ca} = kA\left( {1 - \frac{IAP}{{K_{eq} }}} \right) $$

Here R_Ca_ is the rate of calcite dissolution (mol/s), A is the reactive surface area (m^2^). The ion activity product (IAP) is defined as $a_{{{\text{Ca}}^{2 + } }} a_{{{\text{CO}}_{3}^{{2{ - }}} }}$, and K_eq_ is the equilibrium constant. The IAP/K_eq_ measures the distance from equilibrium. If IAP is lower than K_eq_, the water is under saturated and calcite dissolves; if IAP is higher than K_eq_, the system is over saturated and calcite precipitates. The equilibrium constants are from the standard EQ 3/6 geochemical database [78].

#### Ion exchange

Ion exchange is represented as follows [79, 80]:

4 $$ uBX_{v} \left( s \right) + vA^{u + } \left( {aq} \right) \Leftrightarrow vAX_{u} \left( s \right) + uB^{v + } \left( {aq} \right) $$

Here (aq) and (s) refer to aqueous and exchanged phases, respectively; X^−^ denotes negatively charged exchange sites occupied by cations A^u+^ and B^v+^ of charge *u* and *v* for A and B, respectively. Ion exchange reactions are commonly calculated through the Vanselow convention using cation mole fractions on the exchange sites [81]. The overall cation exchange capacity was calculated based on volume fraction and surface area of clay minerals including muscovite, illite, kaolinite, clinochlore-14A and daphnite-14A. The selectivity coefficients in Table 3 indicate cation affinity to solid surface. The species Ba and Sr have higher affinity than Ca and Mg, which in turn have higher affinity than Na and K. This means that under similar concentration conditions, Ba and Sr tend to be exchanged onto clay surface first before Ca, Mg, and K. The very high Na concentration in Marcellus Shale waters also induces the exchange of Na onto solid surface compared to Ca and Mg.

#### Quantification of release impacts

We define several terms to quantify release impacts on natural water composition. The maximum concentration in receiving waters during release, C_max_, quantifies the magnitude of the impacts. The residence time τ_r_ is calculated by the domain length divided by the natural water flow velocity; it quantifies the time scale at which a non-reactive species stays in the domain of interest. The recovery time, τ_recovery_, is the time scale for each species to return to within 5% difference from its original concentration. Because different species involve different types of water–rock interactions (e.g., mineral precipitation versus ion exchange), this time scale can vary drastically among species. The relative recovery time τ_rr_ is defined as the ratio of τ_recovery_ over τ_r_. The τ_rr_ quantifies the time duration (relative to residence times) that the released chemical species still remain in the simulation domain. All these terms are calculated based on modeling observations from output of numerical experiments. Note that τ_rr_ is a dimensionless number and its value is not constrained to the length or time scale of the calculation domain. The τ_rr_ is similar to the concept of effective retardation coefficient and is species specific [53]. For instance, the retardation factors of Ba and Sr are 111 and 60, respectively under neutral condition [82]. The cations generally follow the retardation sequence of Mg<Ca<Sr<Ba [83, 84]. A higher affinity to solid surface leads to a larger retardation and therefore a slower movement, longer residence time and ultimately longer τ_rr_ and memory.

## Results and discussion

“Controlling processes in the S aquifer” section focuses on understanding processes that control transport and fate of major species in the S aquifer. “Effect of release characteristics in the S aquifer” section assesses the role of release rates. “Effect of receiving water bodies” section compares reactive transport of major species under different release rates under different natural water conditions.

### Controlling processes in the S aquifer

Here we examine the spatio-temporal evolution of major species after release into the S aquifer under four scenarios of increasing process complexity: a case including only mixing without any reactions (“MIX”), a case including mixing and mineral dissolution/precipitation (“MIX + DISS/PPT”), a case with mixing and ion exchange without mineral dissolution/precipitation (“MIX + IEX”), and a case including mixing, mineral dissolution/precipitation, and ion exchange (“MIX + DISS/PPT + IEX”). The release occurred from day 10 to day 25 at the rate of 1.11 × 10^−7^ m^3^/s in all cases. Before the release accident, initial water–rock equilibria are established by continuously injecting natural waters into the simulated domain until their compositions are stabilized.

#### Temporal evolution at the release point

##### Br and Cl

**Fig. 2:**
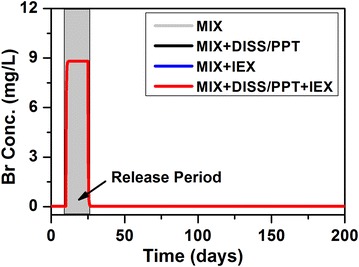
Evolution at the release point for Br under four scenarios. All *four color lines* overlap. The *grey shaded* zone represents the release period. Due to its non-reactive nature, the inclusion of different processes does not affect their evolution

The breakthrough of Br and Cl in the four scenarios are the same due to their non-reactive nature (Fig. 2). The concentrations increase upon release and return to background concentration when the release stops. Their concentrations in the MSW are 185.00 and 29,252.00 mg/L, respectively. With the dilution factor of 21.85, the calculated Br and Cl concentrations during release are 8.82 and 1404.00 mg/L, respectively, approximating their MSW concentrations divided by the dilution factor plus the background concentration.

##### Na, Ca, and Mg

**Fig. 3:**
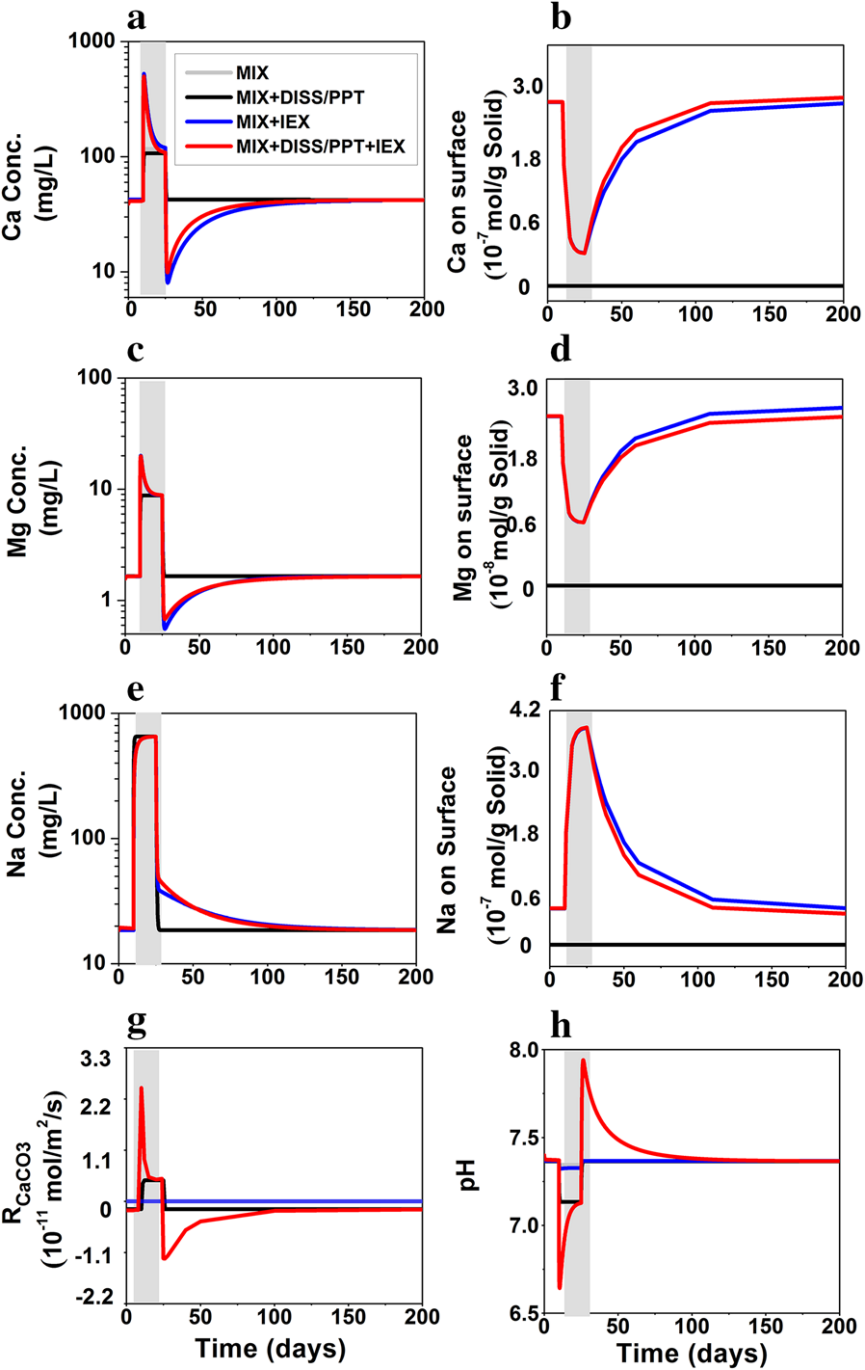
Evolution at the release point for **a** Ca (mg/L) in logarithmic scale, **b** Ca on exchange sites (mol/g solid), **c** Mg (mg/L) in logarithmic scale, **d** Mg on exchange sites (mol/g solid), **e** Na (mg/L) in logarithmic scale, **f** Na on exchange sites (mol/g solid), **g** calcite reaction rate (mol/m^2^/s) (negative indicates dissolution and positive values indicate precipitation), and **h** pH. *Grey line* overlaps with the *black line*

The Na concentration ([Na]) is the highest (13,200.00 mg/L) among the three species in the MSW. The Na exchanges with presorbed Ca and Mg at the solid concentrations of 2.72 × 10^−7^ and 2.55 × 10^−8^ mol/g, respectively. The Ca therefore depends on the mixing with the ground water, dissolution and precipitation of calcite, and ion exchange. In the MIX case, Ca behaves similarly to Cl. The [Ca] in the MIX + DISS/PPT case is lower than that in the MIX case because of calcite precipitation, as indicated by the positive calcite rate in Fig. 3g. In the MIX + IEX case, the [Ca] increases sharply upon release, which echoes the fast Ca decrease on the surface in Fig. 3b and Na increase on the solid phase in Fig. 3f. This indicates that the quick increase is caused by the ion exchange between Na and the presorbed Ca. This desorbed Ca leads to calcite precipitation with sharply increasing rates during release (Fig. 3g positive calcite rates), which decreases aqueous Ca significantly and cause calcite dissolution afterwards (Fig. 3g negative calcite rates). At the time when release stops, the precipitation even draws Ca concentration to below background concentration. The system eventually relaxes back to the original state. Despite the differences in MIX + IEX and MIX + DISS/PPT + IEX cases, similar [Ca] in these two cases indicate the dominance of ion exchange and relatively minor role of calcite dissolution/precipitation when both processes coexist. Compared to the MIX + DISS/PPT case, the increase in [Ca] in the MIX + DISS/PPT + IEX case also leads to much higher calcite precipitation rate during release (Fig. 3a, g).

Similar to Ca, Mg also participates in mineral dissolution/precipitation (clinochlore-14A and dolomite) and ion exchange (Table 3). Compared to Ca, its concentration is about an order of magnitude lower in both background and MSW. Its evolution at the release point resembles that of Ca (Fig. 3c). Although not shown here, dolomite is close to equilibrium while the dissolution rate of clinochlore-14A is in the order of 10^−10^ mol/s. Comparison between the 4 cases show that Mg behaves similarly to Ca and is primarily controlled by ion exchange. The highly elevated Na in MSW leads to massive exchange on the solid surface. After the release stops, Na slowly desorbs, resulting in a long tail for over more than 150 days (Fig. 3e, f). Conversely, Ca and Mg sorb back to the solid (Fig. 3b, d), which results in lower aqueous Ca and Mg concentration when compared to the background concentration and calcite dissolution, as indicated by the negative calcite rates in the MIX + DISS/PPT + IEX case after the release. They eventually return to background concentrations after continuous groundwater flushing and reach equilibrium again.

The original pH values are 7.40 and 6.90 in the S aquifer and MSW, respectively. Values of pH drop upon release in all cases (Fig. 3h). In the MIX and MIX + IEX cases, pH drops slightly and returns immediately to its background when release stops. In the other two cases that involve mineral dissolution and precipitation, pH drops much more significantly during the release, primarily due to calcite precipitation. In the MIX + DISS/PPT + IEX case, because the ion exchange kicks out sorbed Ca and increased aqueous [Ca], the higher calcite precipitation rates lead to more significant pH decrease (Fig. 3g). The calcite dissolution leads to pH increase for an extended period of time until Ca dominates the solid surface again. In general, the pH curve mirrors the shape of calcite rate. The pH values relax back to its background immediately after the release in all cases except the MIX + DISS/PPT + IEX case where pH is mostly controlled by calcite dissolution and precipitation reactions.

##### Ba and Sr

**Fig. 4:**
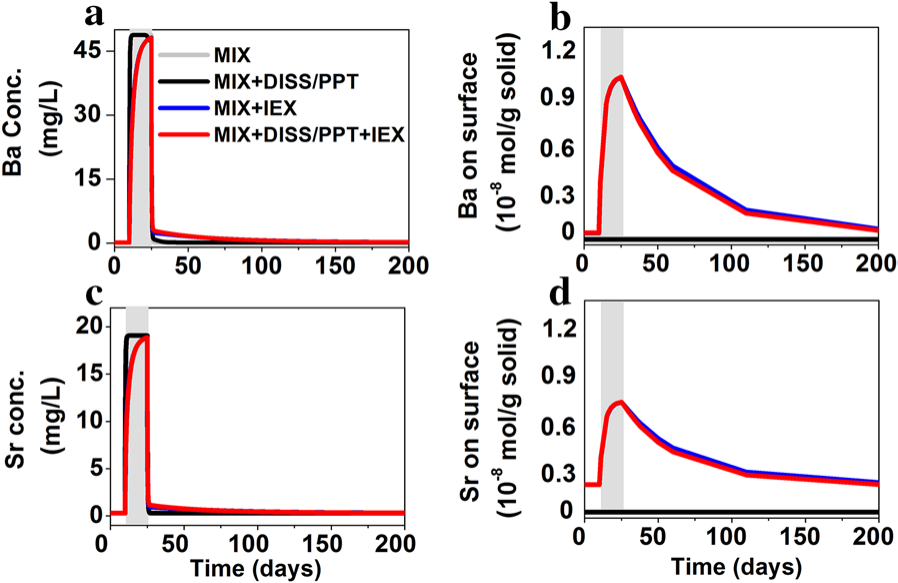
Evolution at the release point for **a** Ba in water (mg/L), **b** Ba on surface (mol/g solid), **c** Sr (mg/L), **d** Sr on surface (mol/g solid). Ion exchange controls concentrations of these species while mineral dissolution and precipitation play a minor role

Barium and strontium exchange with presorbed cations Ca and Mg, leading to decreased aqueous [Ba] and [Sr], and increased aqueous [Ca] and [Mg] in MIX + DISS/PPT + IEX (Fig. 4). After the release stops, Ba and Sr slowly desorb over a longer period of time. Although not shown here, barite and celestite precipitate in negligible rates, indicating the dominant role of ion exchange.

#### Spatio-temporal evolution in the MIX + DISS/PPT + IEX case

Here we examine the spatio-temporal evolution of major species in the MIX + DISS/PPT + IEX case where all processes are included. The release occurs between day 10 and 25 at the rate 1.11 × 10^−7^ m^3^/s.

##### Tracers

**Fig. 5:**
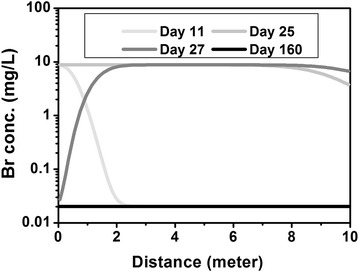
Spatio-temporal evolution of Br concentration in the sandstone aquifer in the MIX + DISS/PPT + IEX case on days 11, 25, 27 and 160. Release starts on day 10 and ends on day 25. The other tracer Cl behaves the same as Br

During release, the [Br] amd [Cl] in the down gradient rapidly increase (Fig. 5). After the release, Cl returns to background concentration starting from the release point. The high concentration zone gradually migrates out of the domain until the system returns to its background.

##### Reactive species

**Fig. 6:**
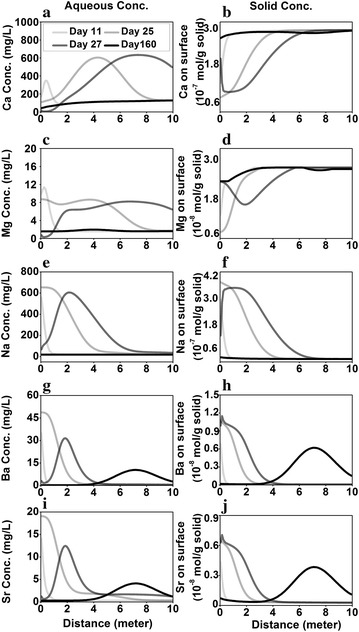
Spatio-temporal profiles of major species in the sandstone aquifer under the MIX + DISS/PPT + IEX scenario on days 11, 25, 27 and 160. *Left column* is for aqueous concentrations (mg/L); *right column* is for concentrations on solid surface (mol/g solid). *Rows* from the *top* to *bottom*: Ca (**a**, **b**), Mg (**c**, **d**), Na (**e**, **f**), Ba (**g**, **h**), and Sr (**i**, **j**)

The five major cations can be categorized into two groups (Fig. 6). Group I includes Ca and Mg (top two rows), both of which are in the MSW and are originally on exchange sites. They are mobilized through ion exchange with cations in the MSW, primarily Na, Ba, and Sr. During release, their aqueous concentration peaks in some zone while the corresponding solid concentration show “valley” of low concentrations. The peaks expand over time during the release. At the end of the incident, their aqueous concentrations are lower than the background concentrations due to their exchange back to the surface. Correspondingly, their solid phase low concentration valleys migrate down gradient slowly over a much longer time scale, long after the release stops on day 25. The depletion zone also becomes wider and shallower due to dispersion as they migrate out of the system.

The Group II species consist of Na, Ba, and Sr, which are abundant in the MSW and exchange with solid surface upon release, displacing Ca and Mg. During release they all show highest aqueous and solid concentrations at the release point, while quickly decrease down gradient. Both peak aqueous and solid concentrations increase over time during release. After the release stops, these cations on the exchange sites gradually become remobilized back into the aqueous phase through ion exchange. Compared to Group I species, Group II species show peaks in both aqueous and solid phases that migrate at similar rates down gradient. The concentration peaks become wider and shallower over time.

##### Quantification of memory indexes from spatio-temporal profiles

The “maximum concentration” C_max_ and the “recovery time” τ_recovery_ can be calculated from the spatial profiles discussed above (Figs. 5, 6). These two measures differ significantly from one species to another. The C_max_ of tracers (Br and Cl) are controlled by the mixing process. After release the system returns to their background after approximately one residence time. For Group I species (Ca and Mg), C_max_ values are higher than those estimated by their dilution factor because they are mobilized from the solid surface during release. For Group II species (Na, Ba, and Sr), their peak concentrations equal to or are lower than those predicted by dilution factor because they exchange onto solid surface. The memory or the time scales of the reactive species are dictated by their affinity to the surface. On day 160, the peak for Na has disappeared, indicating its migration out of the system. In contrast, the peaks of Ba and Sr are approximately at 8 m at that time. As indicated in the ion exchange coefficients in Table 3, the affinity to the surface is Ba/Sr>Ca/Mg>Na. The Ba and Sr therefore migrate out of the system much slower. The τ_rr_ values are 6.79, 9.25, 9.38, 20.09, 18.76 for Na, Ca, Mg, Ba, Sr, respectively. This means that it takes 6.79 residence times to flush out Na, 9.25/9.38 residence time for Ca/Mg, and 20.09/18.76 for Ba/Sr, which are consistent with their affinity to the solid surface. This gradient of time scales consistent with their gradient of the affinity to the solid surface is similar to the chromatographic effects in literature [53].

### Effect of release characteristics in the S aquifer

Three cases were compared with the same release volume of 0.144 m^3^ however at different release rates. The “High” release rate is 1.11 × 10^−7^ m^3^/s for 15 days, the same as the case in the Sect. 4.1. The “Medium” rate is 5.55 × 10^−8^ m^3^/s for 30 days. The “Low” release rate is 1.11 × 10^−8^ m^3^/s for 150 days (Table 4). The corresponding dilution factors are 21.85, 42.70, and 209.54, respectively.

**Fig. 7:**
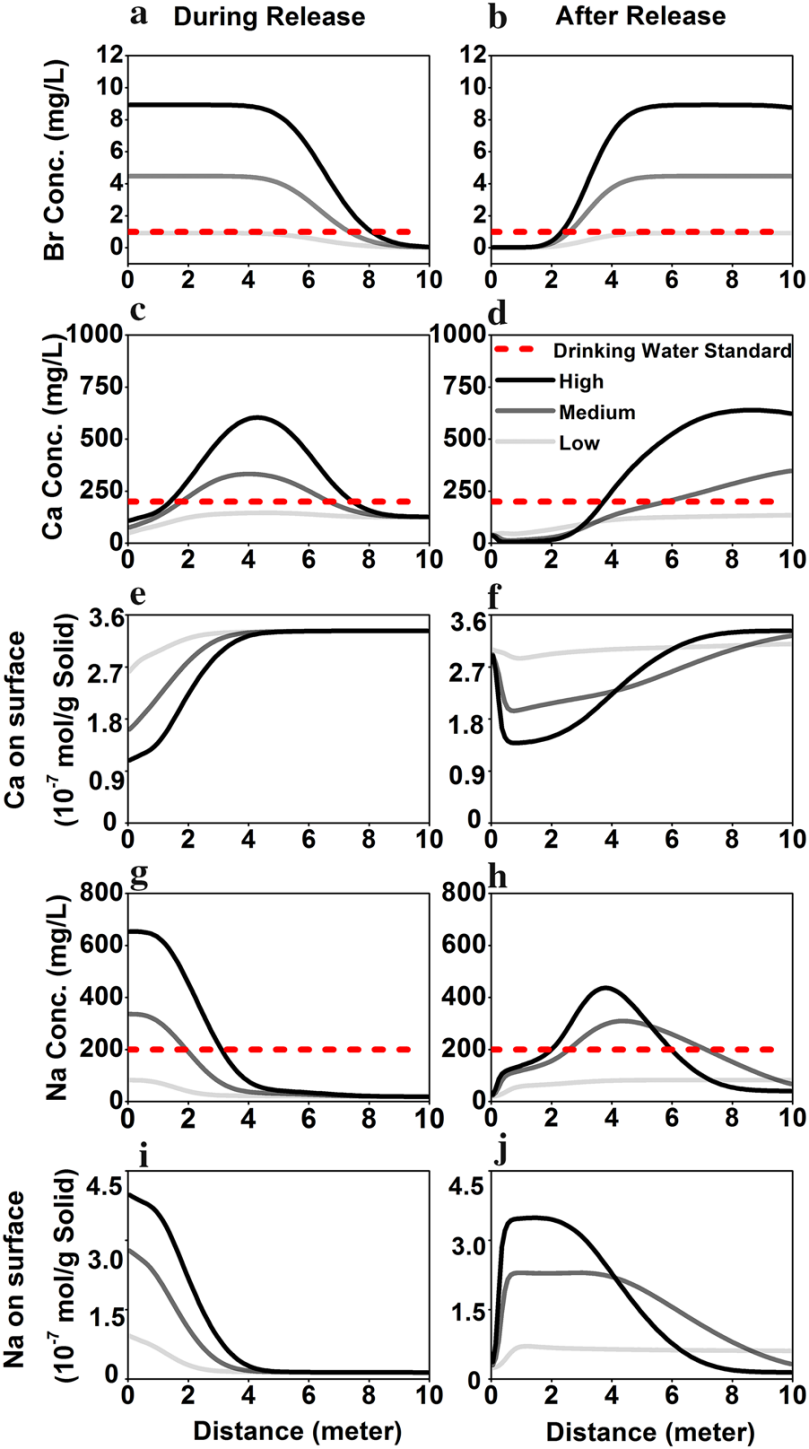
Profiles of Br, Ca, Ca on solid surface, Na, Na on solid surface in the sandstone aquifer during release (*left column*) and after release (*right column*) under the three release cases. The High, Medium, and Low release rates are 1.11 × 10^−7^ m^3^/s for 15 days, 5.55 × 10^−8^ m^3^/s for 30 days, and 1.11 × 10^−8^ m^3^/s for 150 days, respectively. The “during release” curves are on day 10 after the release starts. The “after release” curves are on day 5 after release stops

Figure 7 shows the spatio-temporal evolution for Br (tracer), Ca (Group I), and Na (Group II). In general, the higher release rate, the higher impact on the water chemistry. For the tracers, C_max_ are essentially the MSW concentrations divided by the corresponding dilution factor in each case. For the reactive species, the low release rate leads to much lower aqueous and/or solid concentrations than in the high rate case. In addition, it takes shorter time to flush out Na in the low release rate case and therefore the system recovers sooner.

### Effect of receiving water bodies

The river has the highest flow velocity (2.76 × 10^4^ m/day) compared to the S aquifer (0.20 m/day) and SG aquifers (0.40 m/day). The S aquifer has 21.7 vol% of clay content compared to 0.9% in the SG aquifers and zero in the river. The release occurred at the same high rate of 1.11 × 10^−7^ m^3^/s for 15 days. The dilution factors for the three receiving natural waters are 21.85, 42.70, and 2.87 × 10^6^, for S aquifer, SG aquifers, and river, respectively.

**Fig. 8:**
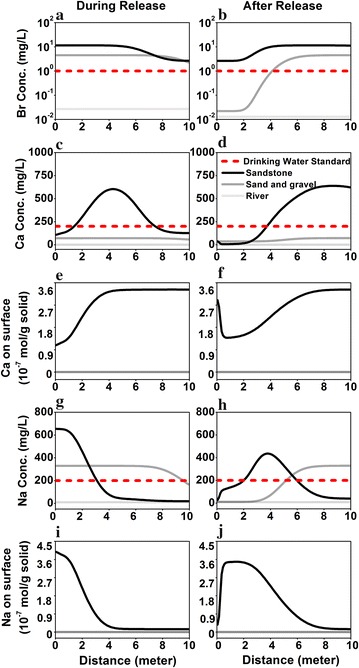
Profiles of Br, Ca, Ca on solid surface, Na, Na on solid surface during release (*left column*) and after release (*right column*) in the sandstone aquifer, sand and gravel aquifer, and river, respectively. The release rate is 1.11 × 10^−7^ m^3^/s for 15 days. The “during release” is on day 10 after the release starts. The “after release” is on day 5 after release stops

Figure 8 shows the effects of receiving water characteristics on the reactive transport of major species. With orders of magnitude higher flow velocity, the river has no memory of MSW—all concentrations are at the background concentration. The MSW however leaves their footprint on the ground water aquifers. The [Br] during the release is lower in the SG aquifers than in the S aquifer due to the higher flow velocity in the SG aquifers. Note that the background [Br] in the two aquifers are also different, with lower [Br] in the SG aquifers. The reactive species behave similarly to the tracers in the SG aquifers because of the low clay content and the lack of ion exchange. The higher dilution factor in the SG aquifers lead to a concentration about half of the maximum [Na] in the S aquifer at the release point, while in the down gradient [Na] is higher in the SG aquifers because negligible ion exchange occurs compared to that in the S aquifer. The [Na] and [Ca] return to the background concentration much faster in the SG aquifers than in the S aquifer.

### Impacts of the release incidents

**Fig. 9:**
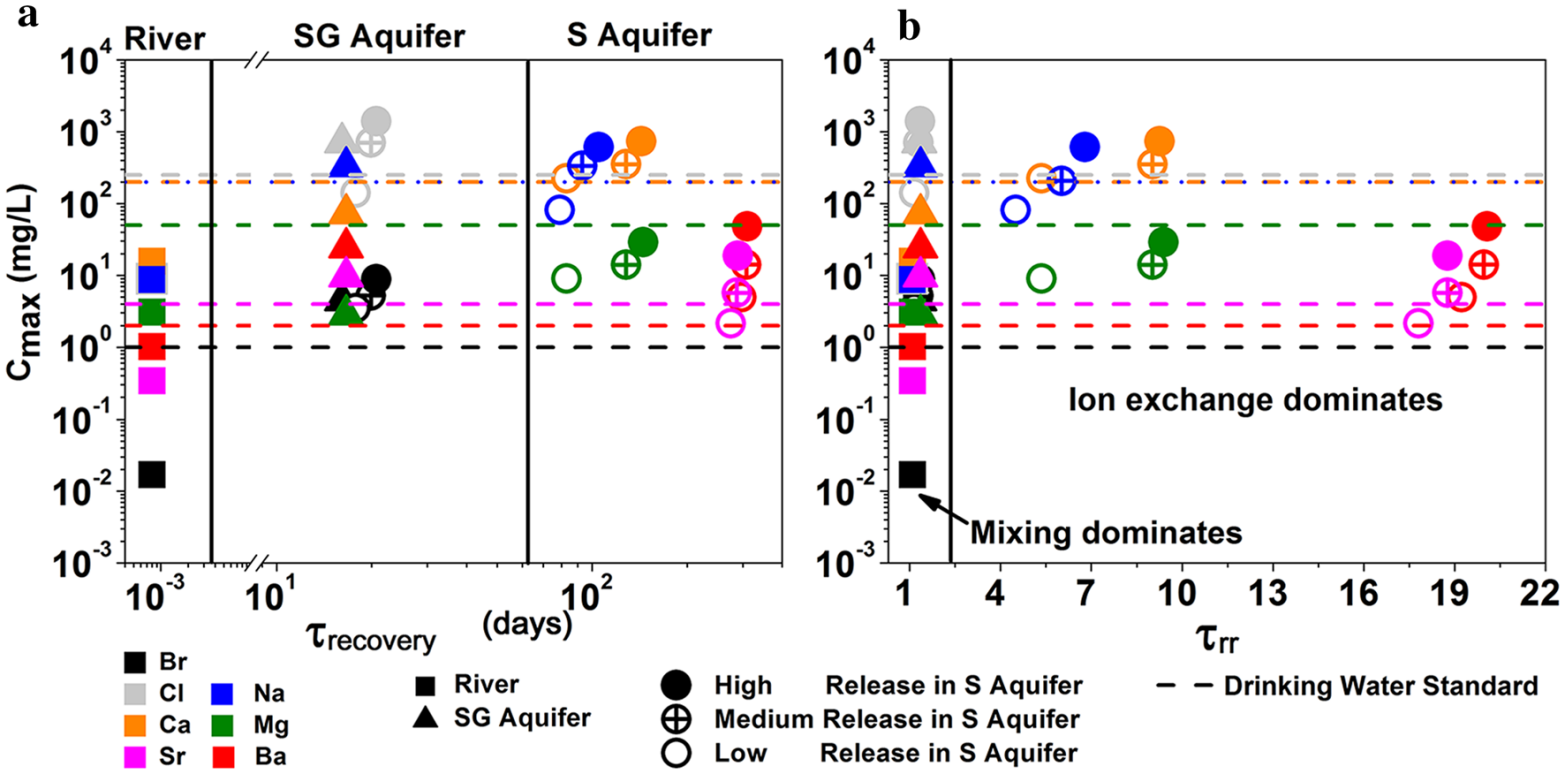
The memory index of natural waters: C_max_ and **a** τ_recovery_ and **b** τ_rr_ of major species in the river (*filled squares*), SG aquifer (*filled triangles*), and S aquifer with high release (*filled circles*), medium release (*crossed circles*), and low release rates (*open circles*). Both are calculated from the modeling output of spatio-temporal concentration evolution. The C_max_ is determined as the maximum aqueous concentration during release. The τ_recovery_ is the time scale for each species to return to within 5% difference from its background concentrations in natural waters. The relative recovery time τ_rr_, calculated as the ratio of τ_recovery_ over τ_r_, is a measure of the time scale that natural waters remember the incident relative to their residence time. Each species is represented by *one color*, with *dashed line* of the *same color* being their drinking water standard. In S aquifer with abundant clay, τ_rr_ values depend on cation affinity to solid surface with τ_rr_ between 5 and 10 for Na, Ca, and Mg, and 15–20 for Sr and Ba

Values of C_max_ and τ_recovery_ quantify the impacts and time scales of release accidents. The numerical experiments indicate that C_max_ of Ca, Na and Cl are 2 orders of magnitude higher than Ba, Sr and Mg (Fig. 9). The river has the lowest C_max_ compared to the groundwater aquifers with all C_max_ values below the drinking water standard [85–87]. In the SG aquifers, all species behave as if they are non-reactive with their C_max_ values proportional to their concentrations in the original MSW. Only the C_max_ of Cl and Na exceed the drinking water standard. In the S aquifer, however, almost all species exceed drinking water standards in the High and Medium release rate cases. In the Low release rate case, only Br and Ba exceed the drinking water standard. The τ_recovery_ values vary by orders of magnitude and depend on specific characteristics of natural waters, release incidents, and individual species (Fig. 9). The S aquifer remembers the incident longer compared to SG aquifers due to the lower flow velocity and higher clay content. The Low release rate case to recover much fast back to the background concentration than the Medium and High cases. Their corresponding relative time scales, τ_rr_, however, vary only from 1.0 to a maximum of about 20 (Fig. 9b). In fact, under all conditions where chemical concentrations are controlled by the mixing process, values of τ_rr_ are close to 1. This includes non-reactive species in all natural waters at all release rates, and reactive species in natural waters with negligible clay content (rivers and SG aquifers). Only in S aquifer with abundant clay, τ_rr_ values depend on cation affinity to solid surface with τ_rr_ between 5 and 10 for Na, Ca, and Mg, and 15–20 for Sr and Ba.

### Discussion

#### Environmental implications

Despite the fact that multiple minerals are involved in dissolution and precipitation, these reactions play a relatively small role compared to ion exchange. This highlights the importance of clay content in determining the time scales and impact of incidental release on natural waters.

The results have interesting implications in understanding reactive transport, monitoring, and detection of contaminants from Marcellus Shale waters in natural water systems. In a controversial example involving unconventional gas wells in a sandstone formation near Pavillion, Wyoming, EPA detected contamination in shallow monitoring wells from 2010 to 2011 [88]. Synthetic organic compounds used in hydraulic fracturing fluids were detected in monitoring wells; [Cl] and [K] were found more than one order of magnitude higher in a monitoring well than the background concentrations. In a second time sampling in the same wells in April and May 2012, some previously detected compounds (e.g., xylenes, toluene) were not found and a number of other compounds have lower concentrations than the previous analysis. As shown in the spatio-temporal figures, there are only certain “time windows” that the signature of MSW can be observed in a specific location, which indicates the ephemeral nature of contamination events and the transient and elusive contamination plumes. This imposes significant challenges to monitoring and detection of groundwater contamination [13].

There have been several cases that discharged MSW were detected in rivers. For example, at the discharge point, [Cl] and [Br] were 6000- and 12,000-fold higher than that in the stream background, both exceeding the drinking water standard [15]. This is a case where the MSW was discharged into river with large volume and therefore the dilution factor of 739 is more than three orders of magnitude lower than that in our model (2.87 × 10^6^). During dry season, low flow rates in rivers lead to lower DF [7], which also increase the possibility of contamination detection. Table 5 shows a few cases where elevated chloride concentrations were reported when MSWs were discharged into river. The DF values in these cases, estimated as the ratio between the flow rates of the river and the discharge rate, vary between 510 and 1246. These values are 3–4 orders of magnitude lower than the DF value in the incidental release case in this work.

**Table 5:** Cases with contamination detected during direct discharge of MSW into rivers [14, 15, 89]

Receiving water body	MSW release rate (×10^−3^ m^3^/s)	River flow rate (m^3^/day)	DF	[Cl] in discharge outlet (mg/L)	Calculated [Cl] in river (mg/L)	Monitored [Cl] in river (mg/L)
Blacklick creek	6.70	432,000	739	80,542.00	107.78	195.00 ± 175.00
Monongahela river	111.10	4,893,000	510	28,879.00	56.62	136.80 ± 2.70
Ten mile creek	11.30	1,223,000	1246	44,915.00	35.84	61.90 ± 2.49

The release rates and flow rates are from literature; DF is calculated as the ratio of the reported river flow rate over the MSW release rate; [Cl] in the discharge outlet are measured values from literature; Calculated [Cl] in river are estimated by dividing the measured [Cl] in the discharge outlet with DF. Monitored [Cl] was directly from literature

#### Limitations

This study is for the specific hydrological and geochemical conditions in Northeastern Pennsylvania in homogeneous systems of one-dimensional 10 m immediately down gradient of the release point where the impacts on natural waters are most significant. This is different from three dimensional natural water systems in reality that have larger dispersion and spreading. As such, the calculation here likely overestimates C_max_ and τ_recovery_ and therefore represents the worst case scenario. However, the quantitative term defined here, especially the relative recovery time τ_rr_, is dimensionless and is not restricted to the length scale of the simulation domain. For example, if the estimated τ_rr_ for a particular species is 5.0, it means that the time needed for recovery is five times of the water residence time. This estimation can be used for systems of different lengths and of flow velocities, because residence times scale with length and flow velocity. As such, τ_rr_ provides the approximation needed for estimating memory or time scales of release incidents in natural waters. In addition, as long as geochemical conditions remain relatively similar, the dominant water–rock interactions are similar.

Here we mainly focus on water–rock interactions of major cations in the MSW without considering redox reactions and biodegradation of organic contaminants that can be present in MSWs. If organic contaminants are present and used by microbe as carbon source, biodegradation reactions will transform organic contaminants into dissolved inorganic carbon, which can increase the concentrations of bicarbonate significantly. Under such circumstances, carbonate precipitation may play a much more significant role, as indicated in literature [49, 67, 90].

This study also considers homogeneous systems. Natural groundwater aquifers are typically layered with heterogeneous distribution of hydrological and geochemical properties [91, 92]. Such spatial heterogeneities have long been reported to cause order-of-magnitude longer tail for non-reactive tracers [93–95] and lower reaction rates [25, 96, 97]. The specific characteristics of different natural water systems, including spatial distribution of clay lenses and layers, therefore, will play a significant role in determining the recovery time of natural waters from incidental release.

In addition, we did not consider the vadose zone processes. Vadose zone processes will affect how much spill volume and chemicals will get into aquifers. However, the major reactive transport processes in natural waters will remain the same and the time scales that the released chemicals remain in aquifer will still be determined by their affinity to the solid surface—this aspect is not going to change whether we include vadose zone processes or not.

## Conclusions

Recent studies on MSWs have mostly focused on evidence linking altered water composition to possible release of Marcellus Shale waters. Process-based understanding and quantification on reactive transport of accidentally released chemicals, however, are largely lacking. Here we use major cations as tracers of release events and reactive transport numerical experiments to illustrate key processes that determine the impacts of accidental release.

The magnitude of the impacts is quantified by C_max_, the maximum observed concentration during release, while the time scale of the impact, τ_recovery_, the required time duration to recover to within (100 ± 5%) of its back ground concentration. We also define a dimensionless number τ_rr_ that is the relative ratio of the τ_recovery_ compared to the residence time of natural waters τ_r_. Our results show that in rivers and SG aquifers with negligible clay content, mixing process controls C_max_ and τ_recovery_ of all species. The dilution factor determines C_max_ while τ_recovery_ approximates the residence time. In clay-rich natural water systems, ion exchange plays a dominant role compared to mineral dissolution and precipitation. The S aquifers with abundant clay selectively remember Sr and Ba for 10–20 residence times due to their higher affinity to clay surface compared to 5–10 residence times for Na, Ca, and Mg. This highlights the importance of clay content in both monitoring and natural attenuation of chemicals from Marcellus Shale waters. This suggests that under otherwise similar conditions, it is more likely to detect contamination in clay-rich geological formations because it takes longer for chemicals to return to its original state in these formations.

This work highlights the usefulness of reactive transport modeling in process understanding and in guiding sampling and monitoring in natural water systems. Findings from this work facilitates prediction of contaminant transport and fate, quantifies impacts of released MSWs in natural waters, and provides insights on risk assessment and strategies for sustainable shale gas development.
